# Hopefulness During COVID-19: Associations With Function, Sleep, and Loneliness in the NHATS

**DOI:** 10.1093/geroni/igab046.2167

**Published:** 2021-12-17

**Authors:** Melissa Hladek, Qiwei Li, Laura Samuel, Brittany Drazich, Thomas Cudjoe, Sarah Szanton

**Affiliations:** 1 Johns Hopkins School of Nursing, Baltimore, Maryland, United States; 2 Johns Hopkins University, Baltimore, Maryland, United States; 3 University of Maryland, Baltimore, Baltimore, Maryland, United States; 4 Johns Hopkins University School of Medicine, Baltimore, Maryland, United States

## Abstract

The associations between hopefulness and function, loneliness, and sleep have not been explored in a nationally representative sample of older adults. Additionally, COVID19 dramatically increased stress burden, potentially influencing these relationships. This study used National Health and Aging Trends COVID19 Supplement data (N=2,894 adults aged ≥ 65 years) to evaluate cross-sectional associations between hopefulness about the future during COVID19 with limitations in activities of daily living (ADLs) using a negative binomial model and one-item sleep and loneliness measures using ordered logistic models. Adjusting for age, race/ethnicity, and education and applying sampling weights, increased hopefulness was associated with better ADLs (b= -0.11, p-value=0.021), less loneliness (b=-0.32, p-value=<0.001), and better sleep (b= -0.27, p-value= <0.001). In the midst of a world-wide stressor, hopefulness was associated with better function and symptoms. This relationship is likely bidirectional and further longitudinal research is needed.

